# Maternal Cardiovascular Emergencies During Pregnancy and the Puerperium: Current Diagnostic Approach and Management

**DOI:** 10.3390/medicina62020401

**Published:** 2026-02-19

**Authors:** Alexandra Arvanitaki, Christos Kalimanis, Athina Nasoufidou, Marios G. Bantidos, Efstratios Karagiannidis, Michail Kalinderis, Nikolaos Fragakis, Barbara Fyntanidou, Ioannis Tsakiridis

**Affiliations:** 1 2nd Cardiology Department, School of Medicine, Faculty of Health Sciences, Aristotle University of Thessaloniki, 54124 Thessaloniki, Greece; 2 Department of Emergency Medicine, School of Medicine, Faculty of Health Sciences, Aristotle University of Thessaloniki, 54124 Thessaloniki, Greecefyntanidou@auth.gr (B.F.); 3 2nd Department of Obstetrics and Gynecology, School of Medicine, Faculty of Health Sciences, Aristotle University of Medicine, 54124 Thessaloniki, Greece; 4 3rd Department of Obstetrics and Gynecology, School of Medicine, Faculty of Health Sciences, Aristotle University of Thessaloniki, 54124 Thessaloniki, Greece; igtsakir@auth.gr

**Keywords:** pregnancy, cardiovascular emergencies, diagnosis, multidisciplinary team management, maternal and fetal outcomes

## Abstract

Physiologic changes during pregnancy, advanced maternal age, and cardiovascular comorbidities have been associated with an increased incidence of cardiovascular emergencies (CVEs) manifesting during pregnancy and puerperium, thereby adversely affecting maternal and fetal morbidity and mortality. When a CVE occurs, prompt and high-quality medical management is essential. However, the early diagnosis and management of CVEs in pregnant women are often challenging, as the initial clinical presentation of many of these conditions may mimic common symptoms of a normal pregnancy, resulting in significant diagnostic delays. Furthermore, the administration of optimal medical or interventional therapy in critically ill pregnant women should be carefully considered, balancing maternal well-being and survival against the potential risks that certain medications and interventions may pose to the fetus. Consequently, treatment decisions should involve a multidisciplinary medical team, comprising cardiologists, obstetricians, emergency physicians, anesthesiologists, neonatologists, and other relevant specialists. This review aims to summarize the current diagnostic approaches and management strategies for the most prevalent CVEs encountered during pregnancy, and explore the challenges faced in diagnosing and treating pregnant individuals compared to the non-pregnant population, emphasizing the differences and knowledge gaps in this area.

## 1. Introduction

Cardiovascular disease affects up to 4% of pregnancies, posing significant risks to maternal health. It has become the leading cause of non-obstetric mortality among pregnant women, highlighting the importance of early detection and management [1]. The incidence of maternal mortality due to cardiovascular etiology has increased dramatically, rising from 3% three decades ago to 15% in recent years [2]. Acute cardiovascular events may occur during pregnancy and the postpartum period and are mainly related to physiological hemodynamic changes, advancing maternal age, a growing number of women with congenital heart disease reaching childbearing age, and preexisting comorbidities [3]. Among the most common causes of sudden maternal deaths are peripartum cardiomyopathy, aortic dissection, acute myocardial infarction, arrhythmias, ischemic heart disease, and coronary artery dissection. Prompt and comprehensive management should be provided by an experienced multidisciplinary Pregnancy Heart Team, with a personalized approach for each case [4]. Given the significantly higher mortality associated with these emergencies, ongoing cardiologic and obstetric vigilance is essential [5]. 

**Figure 1:**
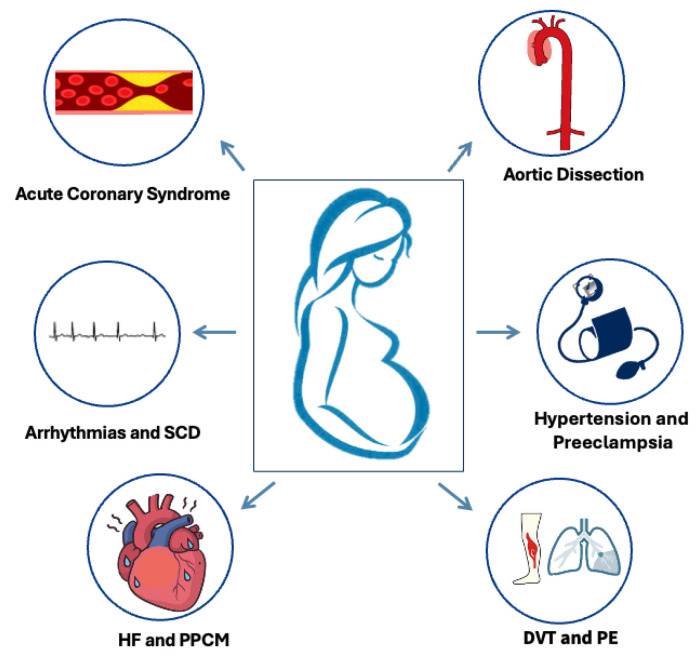
Commonest cardiovascular emergencies in pregnancy and the postpartum. SCD: sudden cardiac death, HF: heart failure, PPCM: peripartum cardiomyopathy, DVT: deep vein thrombosis, PE: pulmonary embolism.

This review aims to summarize current diagnostic approaches and management strategies for the most common cardiovascular emergencies (CVEs) seen during pregnancy and puerperium (Figure 1), explore the challenges faced in diagnosing and treating pregnant individuals compared to the non-pregnant population, emphasizing the differences and knowledge gaps, and discuss future perspectives in the field.

## 2. Methodology

This review presents the current evidence on the diagnosis and management of cardiovascular emergencies during pregnancy and the puerperium. Relevant literature was identified via PubMed, Scopus, and Google Scholar searches covering 2010 to February 2026, using combinations of terms such as pregnancy, puerperium, and cardiovascular emergencies, as well as specific conditions including acute heart failure, acute coronary syndrome, aortic dissection, cardiac arrest, arrhythmias (including supraventricular tachycardia, atrial fibrillation, ventricular tachycardia, and ventricular fibrillation), hypertensive emergencies, preeclampsia, pulmonary embolism, mechanical valve thrombosis, and acute venous thromboembolism. Additional sources were obtained from the reference lists of key publications and major society guidelines. Recent evidence, landmark studies, and expert consensus statements were prioritized. A formal quality appraisal was not performed, as the objective was to provide a concise, clinically oriented synthesis to support multidisciplinary care.

## 3. Physiological Changes in Pregnancy Relevant to Cardiovascular Emergencies

The pathophysiological mechanisms underlying CVEs during pregnancy are predominantly associated with hemodynamic and physiological alterations. From the sixth week of gestation and extending into the second trimester, cardiac output (CO) and stroke volume experience an increase of approximately 30% to 50%, accompanied by an elevation in heart rate of 10 to 20 beats per minute and a reduction in peripheral vascular resistance by 20% to 50% [3,6]. Furthermore, plasma volume expands significantly, while red blood cell counts decrease by 30%, resulting in dilutional anemia. Ventricular function remains preserved; however, both ventricular and atrial dimensions are mildly enlarged. This hemodynamic shift, together with the associated cardiovascular structural adaptations and ventricular wall stretch, may contribute to the occurrence of CVEs during pregnancy, such as acute heart failure (HF), aortic dissection, valvular dysfunction, or tachyarrhythmias, particularly in women with known cardiac disease or predisposing cardiovascular risk factors.

Pregnancy also induces a hypercoagulable state characterized by decreased levels of protein S and increased fibrinogen and other clotting factors. When combined with prothrombotic comorbidities, such as atrial fibrillation, this elevates the risk of thromboembolic events, thereby often necessitating anticoagulation therapy [7].

Furthermore, pregnancy-induced physiological changes—such as altered liver enzyme activity, elevated glomerular filtration rate, and alterations in protein binding—impact the pharmacokinetics of numerous medications, thereby necessitating meticulous dose adjustments [6].

Following delivery, uterine contractions induce a rapid decrease in cardiac output to 15–25% above the normal range, subsequently followed by a gradual decline over the subsequent weeks until normalization by the sixth week postpartum [8]. Consequently, the postpartum period constitutes a critical window that may precipitate cardiovascular events (CVEs), underscoring the significance of careful monitoring and management during this timeframe.

## 4. Cardiovascular Emergencies in Pregnancy

### 4.1. Acute Coronary Syndrome

Acute coronary syndrome (ACS) is a relatively rare complication in pregnancy, with an incidence of 3–8 cases per 100,000 pregnancies [9,10]. However, ACS occurs 3–4 times more frequently in pregnant women than in non-pregnant women of comparable reproductive age and accounts for over 20% of maternal cardiac fatalities [11], with maternal mortality increasing to 5% among pregnant women diagnosed with ACS. The majority of cases present during the third trimester or the puerperium [12].

**Figure 2:**
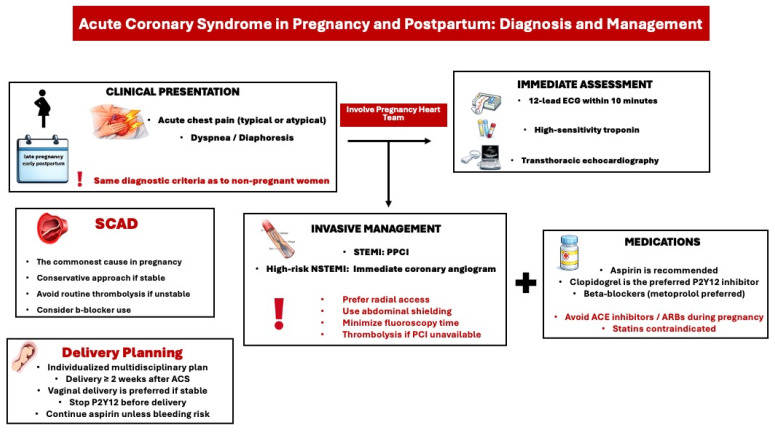
Diagnostic and treatment approach of acute coronary syndrome in pregnancy and postpartum. ACS: acute coronary syndrome, ACE: angiotensin converting enzyme, ARB: angiotensin receptor blocker, ECG: Electrocardiogram, PPCI: Primary percutaneous coronary intervention, SCAD: spontaneous coronary artery dissection, STEMI: ST-segment elevation myocardial infarction, NSTEMI: non-ST-segment elevation myocardial infarction.

The most common cause of pregnancy-related ACS is spontaneous coronary artery dissection (SCAD), which typically manifests in the early postpartum period and is associated with hormonal shifts that alter the synthesis of the coronary arterial wall [13]. It primarily affects the left coronary arteries and should always be considered as a potential diagnosis in women presenting with acute chest pain in the emergency department during the antenatal or postnatal period. Due to the elevated risk of complications related to percutaneous coronary intervention (PCI) such as extending the coronary dissection or iatrogenic dissection with a guidewire (wire entry into the false lumen) or extension of dissection from contrast injection, catheter-induced occlusion of the true lumen (loss of flow after stenting), and the high probability for spontaneous healing of SCAD lesions over time, a conservative management approach is recommended for clinically stable women with SCAD (Figure 2) [4,14]. Overall, the optimal treatment strategy for SCAD during pregnancy remains inadequately defined. Limited observational data suggest that beta-blocker therapy (e.g., labetalol) and strict blood pressure control may reduce the risk of recurrent SCAD. The role of antiplatelet therapy in conservatively managed SCAD remains controversial, although current evidence favors single antiplatelet therapy with aspirin (Level of evidence B) [4,15]. Women with a history of SCAD should be carefully counseled about the risk of recurrence in subsequent pregnancies, as they are considered high risk according to the modified World Health Organization (WHO) classification [4].

The second most common cause of ACS in pregnancy is associated with coronary atherosclerosis [9]. Its incidence rises with advancing maternal age and the presence of comorbidities such as diabetes mellitus (DM), dyslipidemia, chronic hypertension (HTN), prior ischemic heart disease (IHD), family history of coronary artery disease (CAD), and ethnicity, notably the increased likelihood of acute myocardial infarction (AMI) among black women. Additional risk factors encompass gestational hypertension and/or preeclampsia/eclampsia, as well as gestational diabetes. The increasing utilization of in vitro fertilization (IVF) results in more women conceiving at older ages, thereby elevating the risk of cardiovascular risk factors and potential cardiovascular events (CVEs) during pregnancy.

The clinical presentation of ACS during pregnancy or in the postpartum period closely resembles that in non-pregnant women (Figure 2). Common symptoms include sudden chest pain, shortness of breath, acute heart failure, arrhythmias, or cardiogenic shock. According to the latest European Society of Cardiology (ESC) guidelines for managing cardiovascular disease in pregnancy, it is advised to treat pregnant women with ACS the same as non-pregnant women, including diagnostic tests and interventions [4]. When ACS is suspected during pregnancy or postpartum, an ECG, high-sensitivity troponin test, and transthoracic echocardiogram (TTE) should be performed. ECG changes such as ST-segment depression or T-wave inversion can be normal variants in pregnancy. However, an ST-segment elevation in at least two adjacent leads is abnormal and requires urgent management with coronary angiography and PCI. In cases of non-ST-segment elevation ACS, a rise in high-sensitivity troponin indicates myocardial ischemia.

PCI should be performed in cases of atherosclerotic ACS. If PCI is unavailable, thrombolysis may be considered, though it increases the risk of maternal hemorrhage and fetal loss. In cases of myocardial infarction with non-obstructive coronary arteries (MINOCA), conservative management is recommended with further diagnostic tests. After PCI, a dual antiplatelet therapy with aspirin and clopidogrel should be considered. In a conservative approach, monotherapy with aspirin is advised. Antiplatelets should be discontinued several days before planned delivery to prevent epidural hematoma. Overall, recommendations regarding dual antiplatelet therapy after acute coronary syndrome during pregnancy and the duration of anticoagulation are based on limited evidence and are largely extrapolated from general population ESC guidelines (Level of Evidence C). Consequently, management should be individualized, relying on clinical judgment and multidisciplinary decision-making, with careful balancing of maternal and fetal risks. Vaginal delivery is the preferred method if the patient is clinically stable with preserved ventricular function, and delivery should be delayed for at least 2 weeks after an ACS. Finally, there is an evidence gap regarding the necessity and safety of statins in pregnant women with established cardiovascular disease.

### 4.2. Aortic Dissection

Aortic dissection during pregnancy and postpartum is an infrequent yet potentially life-threatening condition. It is predominantly observed in women with thoracic aortic disease, notably those diagnosed with Marfan syndrome [16]. Risk factors encompass an enlarged aortic root exceeding 40 mm, bicuspid aortic valve, aortic coarctation, elevated body mass index (BMI), tobacco use, diabetes mellitus, renal insufficiency, advanced New York Heart Association (NYHA) functional class, preeclampsia or eclampsia, and connective tissue disorders such as Ehlers–Danlos syndrome, Marfan syndrome, and Loeys-Dietz syndrome [4]. Furthermore, physiological alterations associated with pregnancy augment the risk in susceptible women, as elevated estrogen levels may compromise the integrity of the aortic wall. Conversely, an increase in blood volume and afterload resulting from aortocaval compression further exacerbates this risk.

Data from the European Registry of Pregnancy and Cardiac Disease (ROPAC II) indicated that there were only four instances of aortic dissection occurring around the time of delivery and in the early postpartum period among 189 women diagnosed with thoracic aortic disease [17]. No mortalities were reported for either maternal or neonatal cases. Moreover, no complications related to pregnancy were observed in women with a prior history of dissection. The ROPAC III registry documented an incidence rate of 3.5% for aortic dissection (comprising three cases during pregnancy and three cases postpartum) among 176 pregnancies in women with an aortic pathology [16]. Most of these patients had Marfan syndrome and had undergone aortic surgery before pregnancy. Although no maternal or neonatal mortalities were reported, there were six fetal deaths within a cohort of 13 women with aortic pathology who experienced major adverse cardiovascular events.

**Figure 3:**
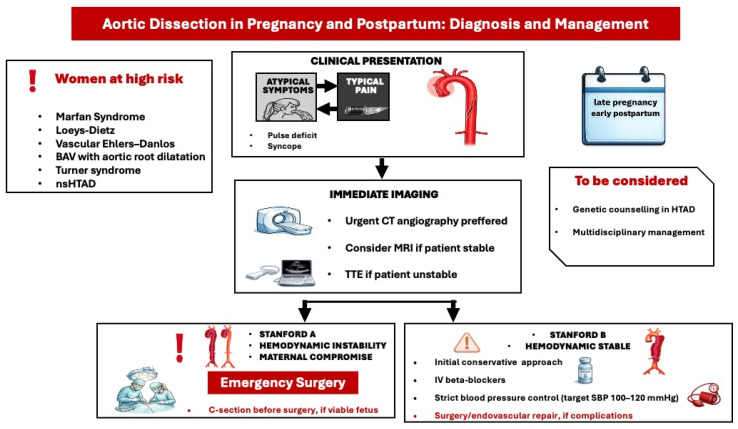
Diagnostic and treatment approach of acute aortic dissection in pregnancy and postpartum. BAV: bicuspid aortic valve, nsHTAD: non syndromic heritable thoracic aortic disease, CT: coronary angiography, MRI: Magnetic resonance imaging, SBP: systolic blood pressure, TTE: transthoracic echocardiography.

Symptoms of aortic dissection may vary from atypical signs, such as vomiting, to severe manifestations, including acute chest pain and syncope. Computed Tomography (CT) angiography (CTA) is considered the preferred imaging modality for suspected aortic dissection due to its high sensitivity and specificity (Figure 3) [18]. During the acute phase of dissection, it is imperative to lower blood pressure with intravenous beta-blockers (e.g., labetalol). Cardiac surgery may be considered during pregnancy when conservative and medical therapies have been ineffective, particularly in scenarios that threaten the maternal life [4]. The decision regarding the acute management of an aortic dissection should be made by a multidisciplinary team comprising experts in aortic and pregnancy-related cardiology. Most type A aortic dissections are managed surgically via aortic replacement. The ESC guidelines recommend that delivery, typically via cesarean section, should be performed before cardiac surgery once the fetus is deemed viable, taking into account factors such as gestational age, comorbidities, and the capabilities of neonatal care (Figure 3) [4]. All recommendations are based on expert opinion, given the lack of strong evidence.

Preventing aortic dissection in women with thoracic aortic disease during pregnancy and the postpartum period remains fundamentally crucial. According to recent ESC guidelines, women diagnosed with aortic disease should be provided with comprehensive counseling concerning the risks of aortic dissection during both pregnancy and the postpartum phase [4]. An expanded multidisciplinary Pregnancy Heart Team must deliver pre-conception counseling to women with a prior history of aortic dissection or surgical intervention, addressing the heightened risks while considering factors such as genetic variant presence and type, aortic morphology, growth rate, and the underlying etiology of the dissection. Before conception, it is recommended that women with known or suspected aortic pathology undergo a computed tomography (CT) or cardiovascular magnetic resonance (CMR) scan of the entire aorta. For pregnant women with aortic disease who present at least an intermediate mWHO cardiovascular risk level, serial echocardiography is advised for ongoing monitoring. Strict and individualized blood pressure management should be maintained in pregnant women with known aortic dilation, a history of dissection, or pathogenic/likely pathogenic (P/LP) genetic variations associated with aortic disease. In cases involving women with Marfan syndrome and other heritable aortic disorders, the use of beta-blockers during pregnancy and postpartum is recommended, accompanied by rigorous monitoring of fetal growth. Beta-blockers were found to be associated with less aortic root growth during pregnancy in 20 patients with Marfan syndrome [19].

### 4.3. Acute Venous Thromboembolism

Venous thromboembolism (VTE) represents a serious and potentially fatal complication that can occur during pregnancy and the postpartum period [20]. The primary manifestations include deep vein thrombosis (DVT) and pulmonary embolism (PE). The incidence of pregnancy-associated VTE is approximately 1.2 per 1000 pregnancies, with a pooled VTE case fatality rate of 0.68% [21]. The VTE risk is highest in the third trimester and in the first 6 weeks postpartum. Possible pathophysiologic mechanisms include pelvic venous and inferior vena cava compression by the gravid uterus, venous stasis, vascular dysfunction, and alterations in coagulation pathways (procoagulant activity) [22]. A formal assessment of VTE risk factors is recommended before or in early pregnancy. In women with risk factors for VTE, thromboprophylaxis with LMWH during pregnancy and the postpartum period is recommended by recent guidelines [22]

#### 4.3.1. Deep Vein Thrombosis

**Figure 4:**
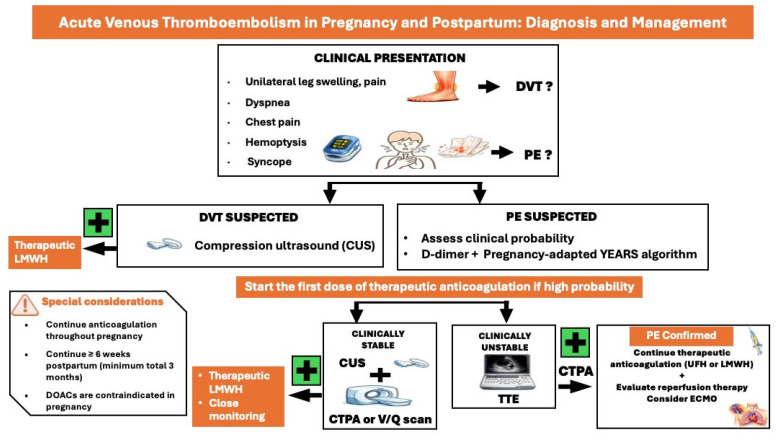
Diagnostic and treatment approach of acute venous thromboembolism in pregnancy and postpartum. CUS: compression ultrasound, CTPA: computed tomography pulmonary angiography, DOACs: direct oral anticoagulants, ECMO: extracorporeal membrane oxygenation, DVT: deep vein thrombosis, LMWH: low molecular weight heparin, PE: pulmonary embolism, TTE: transthoracic echocardiography, UFH: unfractionated heparin.

Physiological signs and symptoms during pregnancy, such as peripheral edema or lower joint pain, may complicate diagnosis. Clinical signs and symptoms suggestive of DVT include pain and swelling in an extremity, presenting with a difference in calf circumference of ≥2 cm. The LEFt criteria (L = Left, symptoms in the left leg; E = Edema, calf circumference difference ≥2 cm; Ft = First trimester of presentation) may be applied to identify women at low risk for DVT (Figure 4) [4].

In pregnant women with a high suspicion of acute DVT, immediate diagnostic workup should be performed. Compression ultrasonography (CUS)—compression ultrasound of infrainguinal veins and ultrasound of iliac veins- is the primary diagnostic modality. In cases of inconclusive results, repeating CUS tests on days 3 and 7 or the utilization of venography or magnetic resonance imaging (MRI) are recommended; a high negative predictive value of CUS of 99.5% (95% confidence interval [CI], 96.9–100%) has been demonstrated [23].

Administration of empiric LMWH is indicated if diagnostic delay is expected and clinical suspicion of DVT is high [20]. Therapeutic dose of Low Molecular Weight Heparin (LMWH) based on early pregnancy body weight is the preferred treatment approach when DVT is confirmed [20]. Consultation with a Pregnancy Heart Team, which should include a vascular specialist and a hematologist, is proposed [4].

#### 4.3.2. Pulmonary Embolism

Clinical signs and symptoms of PE in pregnancy and the postpartum do not differ from those of the general population, including chest pain, dyspnea, hemoptysis, syncope, and cardiogenic shock. Hemodynamic stability is key in the diagnostic and treatment approach of pregnant women suspected of PE (Figure 4) [4,24].

In clinically stable women, the use of adapted D-dimer thresholds in combination with the YEARS criteria (1, clinical signs of acute DVT; 2, haemoptysis; 3, PE is the most likely diagnosis) allow a significant reduction in the need for computed tomographic pulmonary angiography (CTPA) in pregnant women; if YEARS criteria are present, D-dimers should be <500 μg/L to exclude PE, while if YEARS criteria are absent, D-dimers should be <1000 μg/L to exclude PE [25]. If D-dimers are positive, venous compression ultrasound should be performed [4]. In case of a positive result, PE is confirmed without the need for CTPA ventilation/perfusion (V/Q) scan. If venous ultrasound is negative or inconclusive, a CTPA is required to confirm or exclude the diagnosis if clinical suspicion is high. In clinically stable pregnant women with a suspicion of VTE, therapeutic anticoagulation with LMWH should be commenced immediately, even before imaging, until the diagnosis of VTE is either excluded or confirmed.

In hemodynamically unstable women highly suspected of pregnancy-related PE, TTE should be performed to assess right ventricular (RV) dysfunction [4]. In cases of normal RV function, PE is unlikely, and other causes of instability should be investigated. Women with RV dysfunction should undergo CTPA to confirm PE diagnosis. Hemodynamically unstable women with confirmed PE diagnosis are considered at high risk for mortality according to the Pulmonary Embolism Severity Index (PESI) proposed in the 2019 ESC Guidelines for the diagnosis and management of acute pulmonary embolism [24,26]. A catheter-based reperfusion strategy or systemic thrombolysis should be considered after thorough consultation with a specialized multidisciplinary team. In unstable pregnant women with PE, unfractionated heparin (UFH) instead of LMWH may be used in the initial phase of therapeutic anticoagulation. In non-high-risk cases, LMWH in a therapeutic dose, based on the early pregnancy body weight, is usually administered. Vena cava filters should be considered only in cases of recurrent VTE or contraindications to anticoagulation, following multidisciplinary discussion.

Pregnant women receiving a therapeutic dose of anticoagulation need a planned delivery with prior discontinuation of LMWH to prevent spontaneous delivery under full anticoagulation [4]. The duration of therapeutic anticoagulation after pregnancy-related VTE should be at least 3 months overall and at least 6 weeks postpartum [20]. A recent comprehensive review of the guidelines on PE from several scientific societies identified a few discrepancies in the dosage of anticoagulant drugs, indications of anticoagulation monitoring, appropriate management of anticoagulants related to neuraxial anesthesia, and indications for thrombophilia testing following a VTE event [20].

### 4.4. Mechanical Valve Thrombosis

As pregnancy is a hypercoagulable state, women with mechanical valves face a 5–10% risk of prosthetic valve thrombosis during pregnancy, which is associated with increased maternal and fetal mortality [4,27,28]. A mitral prosthetic valve was identified as a predictor for valve thrombosis in a large European cohort [27]. Furthermore, the use of LMWH over vitamin K antagonists (VKA) during the second and third trimesters has been associated with an increased risk of thromboembolic complications [27]. The quality of anticoagulation monitoring in women treated with LMWH cannot be reliably assessed. Although weekly anti–factor Xa monitoring is recommended, optimal target levels remain uncertain, including whether peak or trough measurements best predict thrombotic protection [29]. Evidence comparing vitamin K antagonists and LMWH is limited, as no randomized trials are available and data are largely derived from observational registries.

Clinical presentation includes symptoms and signs of valve obstruction (such as symptoms and signs of acute heart failure, new or worsening murmur, absent prosthetic valve sounds, or syncope) or thromboembolic events [30], which should raise suspicion of acute valve thrombosis in pregnant women with a mechanical valve. Urgent assessment is required. TTE is the initial screening tool, followed by a transesophageal echocardiogram (TOE) in clinically stable women if TTE results are inconclusive. Valve appearance, leaflet mobility, presence of thrombus, and valve gradients can be accurately evaluated in the majority of cases [28]. If the diagnosis remains uncertain, fluoroscopy or cardiac CT may provide additional insights into leaflet mobility and the opening/closing angles of the valve, with minimal radiation exposure to the fetus.

The management of mechanical valve thrombosis in pregnancy is similar to that in non-pregnant women. In clinically stable women without a valve obstruction, anticoagulation optimization with UFH and establishment of a therapeutic INR with a VKA are usually sufficient. Thrombolysis may be an option if there is clinical deterioration despite adequate anticoagulation, and surgery is not immediately available and in right-sided prosthetic valve thrombosis. When obstruction or severe regurgitation of the prosthetic valve is diagnosed, urgent surgery is required. There is a clear survival benefit for the fetus without increasing maternal mortality if cardiac surgery is performed after caesarean section. A Pregnancy Heart Team meeting should take place to discuss the best therapeutic approach based on the type of valve involved, hemodynamic stability of the mother, and gestational age [4].

### 4.5. Hypertensive Emergencies

Hypertensive disorders in pregnancy are the most frequent cardiovascular manifestation in pregnancy, complicating 5-15% of all pregnancies, and include chronic (preexisting) arterial hypertension [31], gestational hypertension (new-onset arterial hypertension after the 20th week of gestation), and preeclampsia, characterized as new-onset hypertension occurring after 20 weeks of gestation (or postpartum) and at least one maternal organ dysfunction including renal dysfunction, proteinuria, liver dysfunction, low platelets, hemolysis, disseminated intravascular coagulation, pulmonary edema, neurological complications (convulsions, headaches, hyperreflexia, visual disturbances, stroke), or adverse fetal outcomes (fetal growth restriction, abnormal umbilical artery Doppler wave or stillbirth) [32]. Arterial HTN in pregnancy is defined as systolic blood pressure ≥140 mmHg or diastolic blood pressure ≥90 mmHg, confirmed on two occasions at least four hours apart [4]. A blood pressure of ≥160/110 mmHg is considered severe hypertension.

Hypertensive disorders in pregnancy are highly associated with increased maternal, fetal, and neonatal morbidity and mortality [33,34]. Therefore, prompt identification and management are required. Personal history of hypertensive disorder in a previous pregnancy, chronic kidney disease, antiphospholipid syndrome, or other autoimmune disease, type 1 or 2 diabetes, chronic hypertension, and use of assisted reproduction techniques are considered as high-risk factors for preeclampsia, whereas primiparity, advanced maternal age (≥40 years old), interpregnancy interval greater than 10 years, maternal obesity, and family history of preeclampsia are considered moderate risk factors by all guidelines [32]. Eclampsia is defined as the new onset of seizures or coma in a pregnant woman with preeclampsia [4].

There is controversy among the guidelines regarding the optimal screening method for assessing preeclampsia risk [32]. A combined screening test that includes assessment of maternal risk factors, uterine artery (UtA) Doppler, maternal blood pressure levels, and serum placental growth factor levels (PlGF) is usually applied. It can effectively detect 75% of cases of preterm preeclampsia and 47% of term preeclampsia, at a false-positive rate of 10% [35]. Screening should ideally be performed in the first trimester, at 11^+0^ to 13^+6^ weeks of gestation, given that the administration of low-dose aspirin (150 mg starting before 16 weeks and up to 36 weeks of gestation) as a preventive strategy is effective in high-risk women, as proven by a large prospective multicenter trial [36].

**Figure 5:**
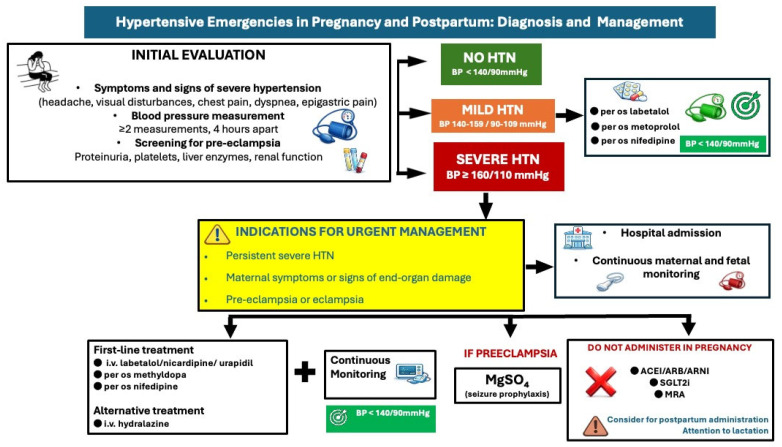
Diagnostic and treatment approach of hypertensive emergencies in pregnancy and postpartum. HTN: hypertension, BP: blood pressure, ACEI: angiotensin converting enzyme inhibitor, ARB: angiotensin receptor blocker, ARNI: angiotensin receptor neprilysin inhibitor, SGLT2i: Sodium-Glucose Cotransporter-2 inhibitors, MRA: mineralocorticoid receptor antagonist.

All women with hypertension in pregnancy should be screened to exclude or diagnose preeclampsia with clinical examination and laboratory testing (Figure 5). Mild gestational or pre-existing hypertension (140–159/90–99 mmHg) without preeclampsia can be medically managed in an outpatient setting with oral methyldopa, labetalol, metoprolol or nifedipine with comparable effectiveness [37,38]. A target of BP: 135/85mmHg has been associated with better pregnancy outcomes [38]; ACE inhibitors and ARBs are contraindicated, and diuretics may negatively affect uteroplacental perfusion.

Hospitalization is necessary in cases of severe hypertension (≥160/110 mmHg) with or without preeclampsia, with administration of intravenous labetalol or nicardipine or oral short-acting nifedipine or methyldopa as first-line medications to control BP [4,39]. Intravenous hydralazine is considered a second-line option. Maternal hemodynamic monitoring (BP monitoring, ECG) and continuous cardiotocographic monitoring should also be considered.

Preeclampsia may require hospital admission, but not all women will require long-term hospitalization (Figure 5); women with severe hypertension or those with neurological, hematological, or cardiovascular complications, liver dysfunction, or renal dysfunction should be hospitalized and strictly monitored. In these cases, administration of magnesium sulfate infusion to prevent eclampsia is suggested, in addition to early delivery (usually after 34 weeks) if there is no clinical improvement [32,40]. A recent randomized controlled trial among 60 pregnant women with severe preeclampsia on the efficacy of antihypertensive drugs (oral nifedipine vs. i.v labetalol vs. i.v hydralazine) demonstrated that oral nifedipine was the most effective drug to reduce BP when single-dose administration was used; it required more doses to reduce BP further. Intravenous hydralazine was the most effective drug when used in up to three doses within an hour, with 20-min intervals [41]. In preeclampsia complicated with pulmonary oedema, intravenous nitroglycerine is recommended. In cases of preeclampsia without severe features, delivery is recommended at 37 weeks [42].

Early postpartum BP control is equally important in these women, as most hypertension-related maternal deaths occur after delivery, due to the increase in systemic BP and result from severe hypertensive complications, i.e., stroke and cardiomyopathy [43]. Nifedipine and labetalol (metoprolol if labetalol is unavailable) are recommended for uncomplicated postpartum hypertension during the first 6 weeks after delivery (Level of evidence C) [4]. For hypertension persisting beyond 6 weeks to 3 months postpartum (late postpartum period), antihypertensive therapy should be initiated according to current guidelines, with consideration of lactation status (Level of Evidence B) [4]. Additionally, lifelong lifestyle and BP screening measures should be taken in women with hypertensive disorders in pregnancy, as they have a higher risk for cardiovascular complications later in life [44].

### 4.6. Acute Heart Failure and Cardiogenic Shock

Acute heart failure (AHF) constitutes the most prevalent complication during pregnancy and the postpartum period, typically presenting after the second trimester or during labor and the early postnatal period [45]. It may manifest de novo in apparently healthy pregnant women (peripartum cardiomyopathy) or as a consequence of underlying conditions such as cardiomyopathy, ischemic heart disease (IHD), congenital heart disease (CHD), severe valvular heart disease (VHD), pulmonary hypertension, and hypertensive disorders of pregnancy. AHF has been associated with an increased risk of maternal mortality and fetal complications, including preterm delivery and neonatal mortality [46,47]. Consequently, early diagnosis and prompt management are imperative [48].

**Figure 6:**
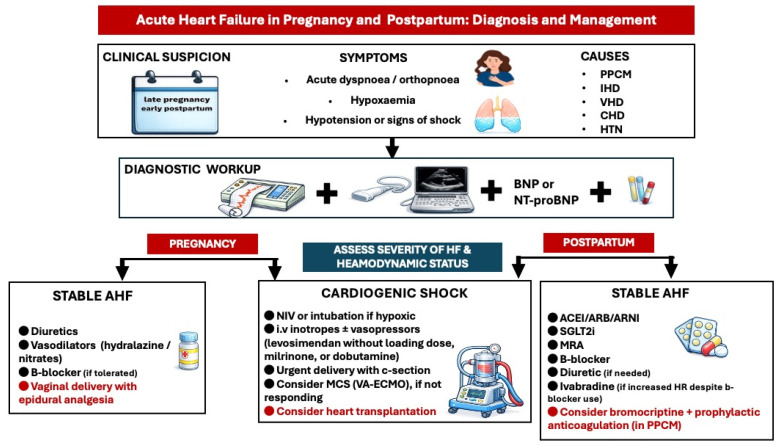
Diagnostic and treatment approach of acute heart failure in pregnancy and postpartum. ACEI: angiotensin converting enzyme inhibitor, AHF: acute heart failure, ARB: angiotensin receptor blocker, ARNI: angiotensin receptor neprilysin inhibitor, BNP: brain natriuretic peptide, CHD: congenital heart disease, HTN: hypertension, IHD: ischemic heart disease, MCS: mechanical circulatory support, MRA: mineralocorticoid receptor antagonist, PPCM: peripartum cardiomyopathy, SGLT2i: Sodium-Glucose Cotransporter-2 inhibitors, VA-ECMO: veno-arterial extracorporeal membrane oxygenation, VHD: valvular heart disease.

Misinterpreting AHF symptoms and signs due to pregnancy-related hemodynamic changes can complicate diagnosis. Acute heart failure symptoms are usually observed in patients with peripartum cardiomyopathy [48]. A thorough personal medical history, physical examination, ECG, brain natriuretic peptides, and transthoracic echocardiography [32] can assist in making the diagnosis [4]. Unfortunately, there are no clear cut-offs for brain natriuretic peptide levels during pregnancy due to lack of validation studies. Pregnant women with AHF require immediate hospital admission; advanced HF management, including on-site surgery and mechanical circulatory support, should be accessible (Figure 6).

In cardiogenic shock, treatment with inotropic agents such as levosimendan, dobutamine, and milrinone and/or vasopressors is recommended (Level of evidence C) [4]. Levosimendan is given as a continuous infusion without an initial loading dose [49]. Mechanical circulatory support—preferably veno-arterial extracorporeal membrane oxygenation (VA-ECMO)—should be considered in cases of severe, refractory cardiogenic shock.

Urgent delivery by cesarean section with combined spinal/epidural analgesia or general anesthesia is advised, as soon as the fetus is viable. Corticosteroids should be administered for fetal lung maturation if the gestational age is less than 34 weeks. Preventing lactation with dopamine receptor agonists may be considered in women with severe heart failure due to the high metabolic demands of lactation.

In the early postpartum phase, intravenous inotropes or vasopressors should be continued in patients with ongoing cardiogenic shock. VA-ECMO may be considered in refractory shock. Evaluation for heart transplantation should be considered for non-responders to mechanical circulatory support [4,50]. Weaning from intravenous inotropes/vasopressors to oral heart failure therapy in postpartum cardiogenic shock should be gradual and guided by continuous hemodynamic monitoring.

Oral diuretics, β1-selective beta-blockers (such as bisoprolol and metoprolol succinate), hydralazine, and oral nitrates can be used to treat women with acute heart failure who are not in cardiogenic shock [13,51]. Because of the potential decrease in uterine blood flow, diuretics (including loop diuretics and thiazides, if necessary) should be used cautiously [52]. However, they may be essential in cases of pulmonary congestion or echocardiographic signs of elevated LV end-diastolic pressure.

It is essential to recognize that most medications utilized in the management of chronic heart failure (HF) within the general population are contraindicated during pregnancy due to potential adverse fetal effects and lack of adequate data to support their use [53]; they include Angiotensin Receptor Neprilysin Inhibitors (ARNIs), Angiotensin-Converting Enzyme (ACE) Inhibitors, Sodium-Glucose Cotransporter-2 (SGLT-2) inhibitors, mineralocorticoid receptor antagonists (MRAs), and ivabradine.

Such medications should be initiated in the early postpartum period, in case of new-onset or worsening heart failure, with consideration given to avoid lactation, if SGLT-2 inhibitors and ARNIs/ACE inhibitors are administered (Figure 6). Treatment initiation, combination strategy, and titration regime should be guided by the severity of ventricular dysfunction and clinical presentation. In severe cases with worsening symptoms, all four drug classes should be introduced at maximally tolerated doses alongside oral diuretics, as extrapolated from general heart failure guidelines (Level of Evidence C) [4]. Bromocriptine, a prolactin-inhibiting agent, effectively suppresses lactation, thus enabling the administration of full-dose heart failure therapy in patients with peripartum cardiomyopathy (PPCM) [18]. Additionally, it further improves ventricular function when added on top of the standard heart failure therapy, by blocking the release of the 16 kDa prolactin fragment that induces ventricular dysfunction [54]. Prophylactic anticoagulation should be contemplated in women receiving this agent to mitigate the risk of thromboembolic events [3].

For heart failure with a reversible cause, treatment should adhere to guideline-recommended protocols, in the late-postpartum period, for a minimum of 12 months following complete recovery of left ventricular (LV) function, after which a careful tapering process should be implemented, with serial echocardiographic assessment. The factors contributing to persistent LV dysfunction and mortality in PPCM are not fully understood. In a prospective cohort of 100 women with PPCM, most recovered by 1 year postpartum [55]. However, 13% had major events or persistent severe cardiomyopathy. Baseline severe LV dysfunction and greater LV remodeling were associated with lower rates of recovery. Further research is needed to elucidate disease mechanisms and to develop novel treatments that improve outcomes and facilitate LV recovery. Women experiencing pregnancy-associated heart failure should be advised regarding the potential risks of recurrence in future pregnancies and offered prompt contraception [19].

### 4.7. Arrhythmias

#### 4.7.1. Supraventricular Arrhythmias

During pregnancy, symptoms such as dizziness, palpitations, or even syncope are relatively common and often benign, as only 10% of them are attributable to cardiac arrhythmias. Supraventricular arrhythmia is the most prevalent arrhythmia observed during pregnancy; although it is frequently benign in nature, it has been linked to an increased risk of maternal morbidity and mortality [56,57].

**Figure 7:**
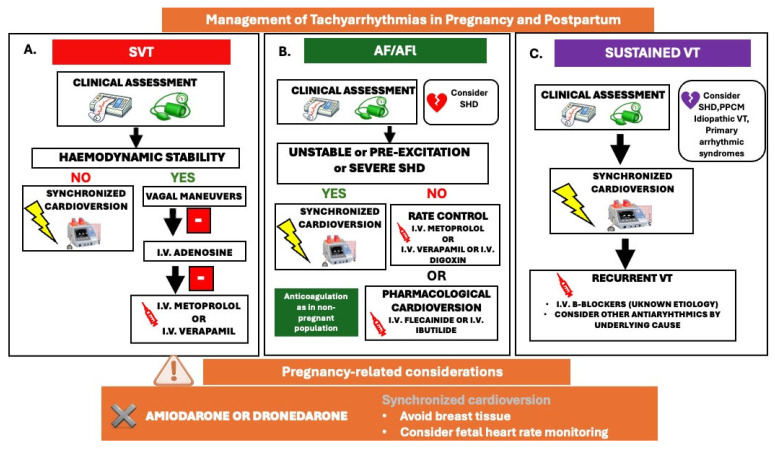
Management of tachyarrhythmias in pregnancy and postpartum; (**A**). SVT: supraventricular tachycardia, (**B**). AF: atrial fibrillation/AFl: atrial flutter, (**C**). VT: ventricular tachycardia. SHD: structural heart disease, PPCM: peripartum cardiomyopathy.

The recurrence rate of supraventricular tachycardias (SVTs) is approximately 50%, with an incidence of 1.3% among women with pre-existing cardiovascular disease [45]. Conversely, the incidence among women without underlying cardiac conditions is considerably lower, and new-onset SVT episodes are infrequent. However, a baseline screening with clinical examination, ECG and echocardiography is essential to exclude underlying cardiac disease [57,58]. According to the latest ESC guidelines, if a pregnant woman exhibits hemodynamic instability, immediate synchronized (CV) cardioversion is advised, with strict fetal heart rate monitoring [4]. In cases where the patient remains hemodynamically stable, vagal maneuvers—such as the modified Valsalva maneuver or carotid sinus massage—may suffice to terminate the arrhythmia. Should these measures prove ineffective, i.v adenosine is recommended for the cessation of SVTs. Furthermore, intravenous beta-1-selective beta-blockers (primarily metoprolol), digoxin, and non-dihydropyridine calcium channel blockers (CCBs) can be employed as secondary treatment options if adenosine fails (Figure 7A) (14).

The incidence of atrial fibrillation (AF) and atrial flutter (AFL) during pregnancy is increasing in association with advanced maternal age (>40 years), multiparity, elevated BMI, arterial HTN, and among women with underlying structural heart conditions, such as CHD, VHD), and cardiomyopathies [59]. According to a recent meta-analysis, the overall estimated incidence of AF among women with structural heart disease during pregnancy was as high as 2.2% with a recurrence rate of 39.2%, while women with no known cardiac disease presented a 0.3% incidence of AF in pregnancy [60]. AF during pregnancy may result in poor maternal and fetal outcomes, like acute heart failure, stroke, stillbirth and preterm delivery [61]. A rapid ventricular response may result in serious hemodynamic consequences for both the mother and the fetus. Electrical cardioversion is considered the first-line treatment for hemodynamically unstable women and for those with severe underlying heart disease [4]. For women who are hemodynamically stable and possess a structurally normal heart, the administration of intravenous anti-arrhythmic drugs (AADs), such as flecainide or ibutilide, is recommended for pharmacological cardioversion. Additionally, i.v beta-blockers or verapamil can be employed for acute rate control if cardioversion is deemed unsafe due to heightened thromboembolic risk (Figure 7B). 

AF/AFl elevates the risk of thromboembolic events, a risk that is further elevated during pregnancy owing to the hypercoagulable state induced by increased coagulation factors, augmented blood volume and cardiac output, and venous stasis [60]. In pregnant women experiencing persistent or permanent AF, the decision to initiate anticoagulation therapy aligns with that for non-pregnant women and is guided by the thromboembolic risk assessed via the CHA2DS2-VASc score [congestive heart failure, hypertension, age ≥75 (doubled), diabetes, stroke (doubled), vascular disease, age 65–74] [4]. Nonetheless, it is essential to note that this score lacks validation within pregnant populations, and its application remains arbitrary. The decision to initiate anticoagulation is generally guided by individualized clinical judgment and multidisciplinary deliberation, with careful consideration of both maternal and fetal risks [58]. Future studies are therefore warranted to estimate the thromboembolic risk among pregnant women with atrial arrhythmias. Direct oral anticoagulants (DOACs) are contraindicated during pregnancy and lactation.

Long-term prophylaxis for supraventricular arrhythmias involves the utilization of beta-1-selective blockers or verapamil in women who do not exhibit pre-excitation on resting electrocardiograms [4]. Flecainide or propafenone are advised for the prevention of arrhythmias in pregnant women diagnosed with Wolff-Parkinson-White (WPW) syndrome. Amiodarone or dronedarone should be avoided during pregnancy (Figure 7).

#### 4.7.2. Ventricular Arrhythmias

The incidence of sustained ventricular tachycardia (VT) in pregnancy is about 1.4% in women with known cardiac disease in Europe, associated with significant morbidity and mortality [62]. Additionally, new onset VT and ventricular fibrillation (VF) occur in approximately 18 per 100,000 hospitalized pregnant women [63].

Recent ESC guidelines in cardiovascular disease and pregnancy recommend immediate electrical cardioversion for all types of sustained VTs in pregnancy, irrespective of hemodynamic stability [4]. Electrical cardioversion is safe and effective throughout all stages of pregnancy, as it does not affect fetal circulation or induce fetal arrhythmias. However, fetal heart rate monitoring should be advised following cardioversion. In cases of recurrent or refractory arrhythmias, intravenous β-blockers or procainamide should be used in women with structural heart disease, while intravenous β-blockers or adenosine should be used in those with idiopathic VT [4] (Figure 7C).

In women with structural heart disease at risk for VT or those with a normal heart, experiencing episodes of idiopathic VTs, oral beta-blockers, at low dose, should be employed as a preventive measure [62]. In instances of refractoriness or contraindications to β-blockers, anti-arrhythmic therapy with flecainide, sotalol, or quinidine is advised, with the selection of the drug based on the underlying cardiac substrate [64]. It is imperative to note that the use of amiodarone, whether for pharmacological cardioversion or prophylaxis, is not recommended during pregnancy and should be restricted to women with refractory or life-threatening arrhythmias that cannot be managed with alternative anti-arrhythmic therapies [4]. If administered, amiodarone necessitates careful monitoring for potential fetal side effects, such as bradycardia or growth restriction.

In cases of necessity, radiofrequency ablation may be performed; however, it is advisable for drug-refractory and poorly tolerated arrhythmias, following the first trimester, to avoid the potential adverse effects associated with prolonged use of antiarrhythmic medications [64]. Non-fluoroscopic electroanatomical mapping navigation systems should be employed in specialized centers.

For pregnant women at high risk of sudden cardiac death (SCD), an Implantable Cardioverter-Defibrillator (ICD) is recommended and can be implanted after the eighth week of gestation without additional risk for the fetus (expert opinion) [65]. The ALARA (As Low As Reasonably Achievable) principle or non-fluoroscopic techniques must be employed to minimize radiation exposure to the fetus.

During delivery, temporary suspension of tachyarrhythmia detection (VT therapy zone should be turned off, or a magnet should be placed if reprogramming is not available) should be considered, particularly when electrocautery is used, with external defibrillation readily available. Neuraxial analgesia is recommended, with careful hemodynamic monitoring, and ICD therapies should be promptly re-enabled immediately postpartum, with device interrogation if therapies were delivered or settings altered [4].

#### 4.7.3. Bradycardia

Bradycardia due to sinus node dysfunction is rare in pregnant women and may usually manifest in those with structural heart disease. If symptomatic bradycardia presents during pregnancy, it is often linked to the supine hypotensive syndrome, caused by uterus compression of the inferior vena cava, and is defined as a decrease of more than 15 mmHg in systolic blood pressure [66]. Mobitz I atrioventricular node block is the commonest type of block during pregnancy, and usually it does not progress.

Management of bradycardia during pregnancy depends on the underlying cause, the presence of structural heart disease or congenital/inherited arrhythmic disease, severity of symptoms, and potential risks to both the mother and the fetus. For women who are asymptomatic and hemodynamically stable, vaginal delivery is safe and poses no maternal or fetal risk [67]. In cases of severe symptomatic bradycardia, intravenous isoproterenol is the recommended first-line drug of choice, with scarce data regarding milk excretion during breastfeeding [4]. The ESC 2025 guidelines adopt the recommendations from the 2021 ESC guidelines on cardiac pacing and resynchronization therapy, as there are scarce data focused on pregnant population [4,68]. If indicated, implantation of a pacemaker during pregnancy is safe when using non-fluoroscopic methods.

Reprogramming of a pacemaker is not required during vaginal or caesarean delivery. In case of electrocautery, bipolar mode is preferred to avoid interference. Nevertheless, in women who are pacing dependent due to sinus node disease, the physiological tachycardic response to hypotension is absent. Consequently, vigilant monitoring for hypotension following neuraxial analgesia or anesthesia and for bleeding is required [65].

### 4.8. Cardiac Arrest

Cardiac arrest during pregnancy and the puerperium has been encountered in about 7–8/100,000 hospitalizations for delivery [69,70,71]. The most common causes are bleeding and amniotic fluid embolism, followed by anesthetic complications and cardiovascular causes (AHF, ACS, arrhythmias, aortic dissection, and PE) [71]. A multicenter retrospective cohort analysis of 87 maternal cardiac arrests demonstrated a 67.8% survival in 30 days with a median post-anesthetic hospital length of stay of 6 days [71]. The advanced life support (ALS) protocol (chest compressions, airway management, defibrillator placement) is similar to the non-pregnant population, with only a few differences that should be considered [72]. If cardiac arrest occurs beyond 20 weeks of pregnancy, left lateral manual displacement of the uterus or left lateral position of the woman is indicated to avoid aortocaval compression [4,72]. Furthermore, i.v line access should be placed above the diaphragm to ensure that the gravid uterus does not obstruct drug administration. No resuscitation drugs should be withheld due to concerns of teratogenicity. In women with preeclampsia who receive magnesium sulfate infusion, it infusion should be discontinued, and calcium gluconate should be administered [4].

If maternal return of spontaneous circulation (ROSC) has not been achieved after 4 min of cardiopulmonary resuscitation (CPR) and the fetus is viable, immediate resuscitative CS should be considered [73]. A recent systematic review and meta-analysis of 176 cases of maternal resuscitative cesarian delivery demonstrated that neonatal survival was more likely when the procedure was performed at a mean time of 10.3 ± 5.5 min [74].

## 5. Challenges and Future Directions

The diagnosis and management of cardiovascular emergencies during pregnancy and the puerperium presents significant challenges that could be attributed to the unique characteristics of pregnancy [3,29,75]: a. the physiologic hemodynamic shifts during pregnancy may be associated with cardiac maladaptation, especially in cases of underlying heart disease; therefore mild hemodynamic changes may lead to severe clinical deterioration, requiring management in the acute setting or may unmask a previously unknown cardiac pathology, b. there is a lack of strong evidence regarding the safety of several antiarrhythmic, antihypertensive, and heart failure drugs during pregnancy and the postpartum period, c. the majority of diagnostic and treatment algorithms in the acute setting have not been validated for the pregnant population.

Therefore, there is an apparent need for prospective multicenter longitudinal studies to address gaps in evidence and provide robust data on the diagnostic and treatment approach of cardiovascular emergencies.

## 6. Conclusions

Acute cardiovascular care during pregnancy and the postpartum period necessitates the involvement of an experienced Pregnancy Heart Team and a well-organized emergency department equipped with specific protocols for each condition. Women in pregnancy and postpartum period experiencing even mild symptoms should seek appropriate medical care and guidance. Intensive medical surveillance throughout gestation and postpartum follow-up can mitigate the risk of acute cardiovascular events. Pre-pregnancy genetic counseling should be offered to women with heritable cardiomyopathies and connective tissue disorders. Therapeutic interventions and pharmacological agents must be carefully tailored, as their indications differ from those for non-pregnant women; many drugs cross the placental barrier or are excreted in breast milk, affect uterine blood perfusion, or pose risks of fetotoxicity and teratogenesis. Consequently, individualized pharmaceutical management is needed. Additionally, maternal psychological support and high-quality postnatal care are essential. As current literature offers limited data, further multicenter studies are warranted in this area. 

## Data Availability

No new data were created or analyzed in this study. Data sharing is not applicable to this article.
